# Compassion in the justification of physician-assisted dying: Gandhi’s non-violence vs. Aristotle’s virtues and vices

**DOI:** 10.1007/s11019-025-10251-0

**Published:** 2025-01-29

**Authors:** Ercan Avci

**Affiliations:** Duquesne University, 600 Forbes Ave, Pittsburgh, PA 15282 USA

**Keywords:** Compassion, Gandhi, Aristotle, Physician-assisted dying

## Abstract

Compassion is an essential phenomenon in the therapeutic relationship, and some use it to justify physician-assisted dying practices. The value of compassion in the relationship between healthcare professionals and patients is undeniable. However, different approaches to its definition and scope can lead to distinct conclusions about the role of compassion in end-of-life interventions. In this context, the paper aims to compare Mahatma Gandhi’s and Aristotle’s views on compassion to explore whether it can be utilized to justify physician-assisted dying. Gandhi’s thoughts on compassion and Aristotle’s standpoint on virtues and vices demonstrate that Gandhi evaluates this concept as a moral duty to relieve intractable suffering, whereas Aristotle relies on balancing all virtues through relevant deficiencies and excesses. Therefore, even though Gandhi’s opinion on compassion can for allow assisted dying interventions, Aristotle’s idea of virtues and vices restricts compassion to a scope that alleviates suffering through available means without causing death.

## Introduction

Relieving pain and suffering has been a primary goal of medicine and the physician’s promise to the patient since the Hippocratic Oath.^1^ This goal is also an important ground for the supporters of physician-assisted dying (PAD).^2^ Euthanasia and physician-assisted suicide (PAS) are two major forms of physician interference, which engender different ethical judgments in death. Albert R. Jonsen and his colleagues list arguments against and in favor of PAD and state the following approach as one of the arguments supporting PAD: “No person should be required to bear disproportionate burdens of pain and suffering, and those who relieve them of such burdens, at their request, are acting ethically, that is, out of compassion and respect for autonomy.”^3^ Tom L. Beauchamp and James F. Childress underscore the function of the virtues of care and compassion, along with the principles of respect for autonomy and beneficence, in the rationale of PAD when there is intolerable suffering.^4^ The emphasis on compassion in PAD prompts a need to appraise the physician’s moral role in the therapeutic relationship in the case of unbearable pain and suffering. The American Medical Association (AMA) considers compassion a necessary component of medical practice through the principle that “[a] physician shall be dedicated to providing competent medical care, with compassion and respect for human dignity and rights.”^5^ Similarly, the World Medical Association International Code of Medical Ethics describes the physician’s primary duty as being “to promote the health and well-being of individual patients by providing competent, timely, and compassionate care in accordance with good medical practice and professionalism.”^6^. Therefore, it is possible to draw a direct connection between compassion and the physician’s moral duty and responsibility, including in the request for PAD. However, understanding or interpreting compassion plays a fundamental role in deciding its function in PAD. For this reason, the paper aims to compare Mahatma Gandhi’s view regarding compassion with Aristotle’s standpoint on virtues and vices to evaluate the possibility of using compassion to justify PAD.

Many scholars consider compassion a virtue that healthcare professionals should possess to understand patients’ needs and approach them appropriately.^7^ Compassion is also a robust moral ground for legitimizing PAD.^8^ Nevertheless, as William E. Stempsey emphasizes, religious views significantly influence people’s stances on PAD.^9^ For instance, Abrahamic religions’ belief that God possesses ultimate power over birth and death precludes the practice of any form of euthanasia or physician-assisted death.^10^ However, regarding religious approaches to PAD, Gandhi, whose political, social, and philosophical thoughts are strongly impacted by his religious views, is an extraordinary figure who submits the utilization of compassion in favor of assisted dying.^11^ In this context, studying compassion through Aristotle’s opinion on virtues and vices and Gandhi’s religion-oriented standpoint can provide a useful insight into the role of compassion in the justification of PAD.

## Compassion

The significance of compassion in medicine is an overwhelmingly accepted phenomenon, but its definitions vary slightly.^12^ Charles J. Dougherty and Ruth Purtilo consider compassion an essential component of the relationship between the physician and patient.^13^ Ramy Sedhom defines compassion as “a deep response to the suffering of another.”^14^ Gyatso Tenzin, the 14th Dalai Lama, deems compassion “not just an emotional response but a firm commitment founded on reason.”^15^ Harvey Max Chochinov describes compassion as “a deep awareness of the suffering of another coupled with the wish to relieve it.”^16^ Constance E. George describes compassion as “an indicator of quality for patients and … an indicator of good character and collegiality among clinicians.”^17^ Additionally, according to Andre Comte-Sponville, compassion is a universal virtue due to its intention that aims to mitigate suffering regardless of the cause of the suffering and because of its scope, which not only covers human beings’ suffering but all beings’ suffering.^18^ David C. Thomasma also considers compassion to be a virtue that generates feelings urging us to help the sufferer.^19^

Based on these different descriptions and approaches, it is possible to highlight three aspects of compassion. First, compassion refers to emotions. The sufferer’s unfortunate circumstances touch others’ feelings and create sympathy for the person. Second, compassion incorporates the wish to help someone in pain or suffering. This point differentiates compassion from sympathy. Third, compassion may be accepted as a moral value, virtue, or characteristic of healthcare professionals in the therapeutic relationship. Even though compassion is a subjective concept, it indicates a desirable characteristic of people practicing healthcare services.^20^

## Compassion in Gandhi’s principle of non-violence

In general, compassion in medicine is deemed a virtue for or a personal characteristic of healthcare professionals without any religious references. Thus, compassion can be acknowledged as a moral value rather than a religious term. However, Mahatma Gandhi completely appraises compassion from a religious standpoint, takes compassion to be “at the very root of religion,” and says, “[r]eligion without compassion is a fraud.”^21^ Ahimsa (non-killing) is an essential component of Hinduism, Jainism, and Buddhism.^22^ Gandhi assesses the broad meaning of compassion via ahimsa and claims that “[t]he very essence of ahimsa lies in burning our anger and in cleansing the soul.”^23^ In other words, Gandhi converts the concept of non-killing into the principle of non-violence and defines the state of non-violence as “an ocean of compassion.”^24^ From this perspective, according to Gandhi, compassion is the most important component of the person’s dharma and a necessary part of non-violence.^25^

Gandhi’s broad framework of compassion can be examined through two sub-categories: Compassion as relieving suffering and compassion as doing good or performing our duty. Gandhi says, “I feel pain when I see others suffer. It is the nature of compassion that whenever one is unable to relieve the suffering of others one suffers unbearably.”^26^ He also expresses that “[t]rue compassion lies in doing what is good or performing our duty even at the cost of inflicting pain on others through our action.”^27^ The former quotation deems compassion to be a strong wish to help the sufferer, and the latter one indicates compassion as acting according to our duties even in the case of causing pain, such as not giving money to your alcohol-addicted child or allowing her/him to drink alcohol. From this perspective, Gandhi’s compassion can be defined in light of these two approaches.

The doctrine of non-violence rejects harming any beings and requires love and compassion toward them.^28^ Therefore, compassion plays an indispensable role in the idea of non-violence. However, this does not mean that Gandhi refuses all forms of inflicting harm or pain. On the contrary, according to Gandhi, under the condition of the following situations, inflicting harm or pain is the requirement of compassion:

“1. We can do something which hurts someone only if it hurts us more than it hurts him.

2. Our motive must be absolutely pure. We should have no other thought in our mind than the welfare of the other person.”^29^

Based on these conditions, harm or pain can be inflicted for the sake of the sufferer’s welfare. Gandhi even takes this permissibility to a level of moral obligation when there is no alternative to relieve suffering. He states,

“I cannot for a moment bear to see a dog, or for that matter any other living being, helplessly suffering the torture of a slow death. I do not kill a human being thus circumstanced because I have more hopeful remedies. I should kill a dog similarly situated, because in its case I am without a remedy. Should my child be attacked by rabies and there was no hopeful remedy to relieve his agony, I should consider it my duty to take his life.”^30^

This quotation demonstrates both aspects of compassion: Helping the sufferer and performing our duty. The goal is to help alleviate suffering, but it is not only an act of kindness or benevolence but also a moral requirement resulting from our duty.

The example of rabies also shows that Gandhi does not have any limitations to the application of compassion. Thus, Gandhi says, “[t]here is religion only to the extent that there is compassion. There can be no limit to compassion.”^31^ Gandhi’s religious view on compassion can be regarded as a supportive argument for the acceptability of PAD practices. Moreover, it is even possible to interpret his standpoint as a moral obligation to implement PAD under the conditions of intractable pain or suffering, pure motivation, and unavailability of alternatives.

## Aristotle’s view on virtues and vices

In *Nicomachean Ethics*, Aristotle considers happiness “the best, noblest, and most pleasant thing in the world” as “an activity of soul in accordance with perfect virtue.”^32^ According to Aristotle, virtue is “a state of character” aiming to find a mean between deficiency and excess “in passions and in actions.”^33^ He claims that virtue can be acquired only by pinpointing the middle of two vices, deficiency and excess, but it is not an easy task everyone can achieve.^34^ Additionally, a mean (intermediate) is only possible for good passions and actions, not intrinsically bad things, like murder, theft, or envy, or excesses and deficiencies.^35^ Aristotle also gives many examples of means with relevant excesses and deficiencies, some of which are as follows^36^:

**Table:** 

Passion/Action	Virtue	Excess	Deficiency
Feelings of fear and confidence	Courage	Rashness	Cowardice
Giving and taking money	Liberality	Prodigality	Meanness
Money	Magnificence	Tastelessness and Vulgarity	Niggardliness
Honor and dishonor	Proper pride	Empty vanity	Undue humility
Anger	Good temper	Irascibility	Inirascibility
Pleasantness in giving amusement	Wittiness	Buffoonery	Boorishness
The remaining kind of pleasantness (exhibited in life in general)	Friendliness	Obsequiousness	Quarrelsomeness and Surliness
Passions	Modesty	Shyness	Shamelessness
Pain and pleasure that are felt at the fortunes of our neighbors	Righteous indignation	Envy	Spitefulness
Pleasures and pains	Temperance	Self-indulgence	Insensibility

Besides these specific examples of virtues and the possible vices of each, Aristotle draws a general framework about how to decide on virtues by asserting that feeling passions and actions “at the right times, with reference to the right objects, towards the right people, with the right motive, and in the right way” depends on “the quality of aiming at the intermediate.”^37^ From this perspective, any passion or action, including compassion, can be appraised in accordance with this view to determine the pertinent state of virtue. However, it should be highlighted that Aristotle’s standpoint on virtues and vices indicates specific limitations to all passions and actions.

## Compassion in PAD: Gandhi vs Aristotle

Compassion is a moral value and requires helping the sufferer. Gandhi deems compassion to be a duty to help and relieve the sufferer’s pain and suffering without any limitation. Even though compassion is a primary component of the principle of non-violence, which refuses to harm or kill any beings and requires showing love to them, under the condition of pure motivation, Gandhi justifies taking the life of a human being who suffers unbearably and has no possibility of remedy. In the light of compassion, Gandhi’s view can be interpreted such that PAD is morally justifiable if the goal is to relieve intractable pain or suffering, if there are no alternatives to relieve the intractable pain or suffering, and if practicing PAD hurts/saddens the practitioner.

Based on Aristotle’s idea of virtues and vice, compassion can be considered a virtue because it is a passion indicating the wish to help the sufferer. However, Aristotle does not acknowledge any passion in a limitless state but claims the necessity to find the intermediate for all passions and actions. Therefore, in the case of regarding compassion as a virtue, its excess and deficiency states should also be reckoned. In the translation of *Nicomachean Ethics* and *The Art of Rhetoric*, the term “pity” is used rather than compassion.^38^ However, according to John Saunders, “pity” in modern translation of Aristotle’s works refers to compassion.^39^ Aristotle defines pity as “a feeling of pain caused by the sight of some evil, destructive or painful, which befalls one who does not deserve it” and describes the person who feels pity as one who is between two extremes: The insolent person, who has no concern about any evil that happens to her/him and the panic-stricken person who is preoccupied with what happens to her/him.^40^ In this view, it is possible to argue that Aristotle uses pity only as a feeling of pain, not a wish to do something to alleviate pain. Thus, in *Nicomachean Ethics*, Aristotle says, “with respect to weakness and infirmity; no one would reproach a man blind from birth or by disease or from a blow, but rather pity him, while everyone would blame a man who was blind from drunkenness.”^41^ From this perspective, a conclusion may be drawn as Aristotle’s utilization of pity in *Nicomachean Ethics* and *The Art of Rhetoric* refers to sympathy rather than compassion because compassion not only indicates a sense of understanding the person’s suffering; it also requests to do or wish to do something to relieve the suffering.

Aristotle elaborates on the virtues and vices of several passions in *Nicomachean Ethics*, but compassion is not one of them. However, based on his general framework of virtues and vices, compassion can undoubtedly be accepted as a virtue. In such a case, the excess and deficiency of compassion should also be reckoned, and fixation might be recognized as the excess, whereas apathy may be contemplated as the deficiency. Fixation refers to the state of feeling responsible for everything and striving to have control over all circumstances. Gandhi’s example of responsibility for taking the life of a person attacked by rabies can signify such a situation because instead of admitting the person’s unfortunate condition/fate, showing patience, and acknowledging God’s decision, assuming the responsibility for ending the suffering at all costs, even by killing, may reveal such an extreme. On the other hand, apathy might show a deficiency of compassion because besides sympathy for the suffering, trying to mitigate it is an essential part of compassion. However, not demonstrating any wish to help the suffering person points out a deficiency.

Compassion, as a virtue that is the intermediate between fixation and apathy, cannot justify PAD practices. Providing a person suffering intolerably with palliative, social, emotional, and spiritual care may be consistent with compassion. However, taking this effort even further by terminating the person’s life might become a vice as an excess of compassion. As a result, since Aristotle defines virtues through certain limits, contrary to Gandhi’s standpoint, according to Aristotle’s view on virtues and vices, compassion cannot be considered a moral justification for PAD interventions.

## Conclusion

Even though the need for compassion in the therapeutic relationship is acknowledged by consensus, its utilization in the justification of end-of-life interventions generates controversial arguments. In particular, the views legitimizing PAD deploy compassion as a primary ground to support assisting people who suffer intolerably to die. Considering compassion a virtue and defining it as a strong feeling of helping the sufferer may lead to a conclusion of alleviating the suffering at all costs. However, examining compassion from the perspective of Aristotle’s thoughts on virtues and vices demonstrates that compassion should indicate an intermediate level, such as an area between apathy and fixation, to be acknowledged as a virtue. In other words, according to a virtue-oriented evaluation, compassion should not be used to justify PAD interventions because it requires helping the person to relieve suffering, but the help should be limited to an intermediate approach (a mean); relieving suffering at all costs would represent an excess. Therefore, rather than describing compassion as a moral duty or responsibility for alleviating suffering at all costs, assessing compassion as an intermediate way to help patients with available medical, spiritual, emotional, and social support may be construed as a virtue ethics-based requirement.

On the other hand, despite the strong religious impact on his political, social, and philosophical views, Mahatma Gandhi accepts compassion as a moral duty and admits it as a basis, under certain conditions, for mitigating intractable suffering at all costs, including assisted dying practices. At that point, Gandhi’s approach is significant, not only because of his endorsement of PAD, but also because of his dissent to religious arguments on PAD.

Additionally, it is generally possible to presume Aristotle’s non-religion-driven thoughts in favor of assisted dying and to assume Gandhi’s religion-based standpoint against PAD practices. However, the paper reveals that based on a compassion-related comparison, both display opposite positions to that general expectation because Gandhi agrees on relieving intractable pain or suffering through all available means in the event of the lack of alternatives and self-interest motivations, whereas Aristotle’s approach to virtues asks us to interpret compassion through an intermediate stance, which demands helping sufferers without killing them or directly causing their death.

## Footnotes

1 National Library of Medicine, “Translation of the Hippocratic Oath,” accessed May 22, 2024, https://www.nlm.nih.gov/hmd/topics/greek-medicine/index.html. - Daniel Callahan, “The Goals of medicine-Setting new priorities,” *Hastings Center Report* 26, no. 6 (1996): 1–27.

2 Sarah Mroz et al., “Assisted Dying around the World: A Status Quaestionis,” *Annals of Palliative Medicine* 10, no. 3 (2021): 3550.

3 Albert R Jonsen, Mark Siegler, and William J. Winslade, *Clinical Ethics: A Practical Approach to Ethical Decisions in Clinical Medicine* (New York: McGraw Hill Education, 2010), 158.

4 Tom L. Beauchamp and James F. Childress, *Principles of Biomedical Ethics*, 7th edition (New York; Oxford: Oxford University Press, 2013), 185.

5 Ercan Avci, “The Goals of Medicine and Compassion in the Ethical Assessment of Euthanasia and Physician-Assisted Suicide: Relieving Pain and Suffering by Protecting, Promoting, and Maintaining the Person’s Well-Being,” *Journal of Palliative Care* 37, no. 3 (2022): 366–371. - AMA, “AMA Principles of Medical Ethics,” accessed May 22, 2024, https://code-medical-ethics.ama-assn.org/principles

6 World Medical Association, WMA International Code of Medical Ethics, accessed on December 31, 2024, https://www.wma.net/policies-post/wma-international-code-of-medical-ethics/.

7 Tom L. Beauchamp and James F. Childress, *Principles of Biomedical Ethics*, 7th edition (New York; Oxford: Oxford University Press, 2013), 37–39. - Andre Comte-Sponville, *A Small Treatise on the Great Virtues*, trans. Catherine Temerson (New York: Holt Paperbacks, 2002), 105-111. - David C. Thomasma and Thomasine Kushner, “A Dialogue on Compassion and. Supererogation in Medicine,” *Cambridge Quarterly of Healthcare Ethics*, no. 4 (1995): 416.

8 Bernard Lo, *Resolving Ethical Dilemmas: A Guide for Clinicians*, 6th edition (Philadelphia: Wolters Kluwer, 2020), 147.

9 William E. Stempsey, “The role of religion in the debate about physician-assisted dying,” *Medicine, Health Care and Philosophy* 13 (2010): 383–387.

10 W. Falk Ze’ev,”Jewish perspectives on assisted suicide and euthanasia,” *Journal of Law and Religion* 13, no. 2 (1998): 379–384. - David F. Kelly, Gerard Magill, and Henk Ten Have, *Contemporary Catholic Health Care Ethics* (Washington D.C.: Georgetown University Press, 2013), 210–218. - Ercan Avci, “A comparative analysis on the perspective of Sunni Theology and Hindu Tradition Regarding Euthanasia: The impact of belief in resurrection and reincarnation,” *Journal of Religion and Health* 58, no. 5 (2019): 1770–1791.

11 K.K. Misra, “Social and Political Thought of Gandhi by J. Bandyopadhyaya: Evolution of the Political Philosophy of Gandhi by B. Bhattacharyya,” *The Indian Journal of Political Science* 31, no. 2 (1970): 187–193. - Joris Gielen, “Mahātmā Gandhi’s view on euthanasia and assisted suicide,” *Journal of Medical Ethics* 38, no. 7 (2012): 431–434.

12 Antonio T Fernando, Katherine Skinner, and Nathan S. Consedine, “Increasing Compassion in Medical Decision-making: Can a Brief Mindfulness Intervention Help?,” *Mindfulness* 8 (2017): 276.

13 Charles J. Dougherty and Ruth Purtilo, “Physicians’ Duty of Compassion,” *Cambridge Quarterly of Healthcare Ethics*, no. 4 (1995): 433.

14 Ramy Sedhom, “Taking Our Oath Seriously: Compassion for Patients,” *AMA Journal of Ethics* 18, no. 1 (2016): 71.

15 Tenzin Gyatso, “Compassion and the Individual,” accessed May 23, 2024, https://www.dalailama.com/messages/compassion-and-human-values/compassion. n.p.

16 Harvey Max Chochinov, “Dignity and the Essence of Medicine: the A, B, C, and D of Dignity Conserving Care.” *BMJ* 335, no. 7612 (2007): 186.

17 Constance E. George, “What Might a Good Compassionate Force Protocol Look Like?,” *AMA Journal of Ethics* 23, no. 4 (2021): 332.

18 Andre Comte-Sponville, *A Small Treatise on the Great Virtues*, trans. Catherine Temerson (New York: Holt Paperbacks, 2002), 105-111.

19 David C. Thomasma and Thomasine Kushner, “A Dialogue on Compassion and. Supererogation in Medicine,” *Cambridge Quarterly of Healthcare Ethics*, no. 4 (1995): 416.

20 Ercan Avci, “The Goals of Medicine and Compassion in the Ethical Assessment of Euthanasia and Physician-Assisted Suicide: Relieving Pain and Suffering by Protecting, Promoting, and Maintaining the Person’s Well-Being” *Journal of Palliative Care* 37, no. 3 (2022): 366–371.

21 Mahatma Gandhi, *The Collected Works of Mahatma Gandhi* vol. 30, p. 106, accessed on May 25, 2024, http://www.gandhiashramsevagram.org/gandhi-literature/mahatma-gandhi-collected-works-volume-30.pdf,

22 Ercan Avci, “A comparative analysis on the perspective of Sunni Theology and Hindu Tradition Regarding Euthanasia: The impact of belief in resurrection and reincarnation,” *Journal of Religion and Health* 58, no. 5 (2019): 1776–1777.

23 Mahatma Gandhi, *The Collected Works of Mahatma Gandhi Vol. 25*, p. 95, accessed on May 25, 2024, http://www.gandhiashramsevagram.org/gandhi-literature/mahatma-gandhi-collected-works-volume-25.pdf.

24 Mahatma Gandhi, *The Collected Works of Mahatma Gandhi Vol. 43*, p. 7, accessed on May 25, 2024, http://www.gandhiashramsevagram.org/gandhi-literature/mahatma-gandhi-collected-works-volume-43.pdf.

25 Mahatma Gandhi, *The Collected Works of Mahatma Gandhi Vol. 47*, p. 29, accessed on May 25, 2024, http://www.gandhiashramsevagram.org/gandhi-literature/mahatma-gandhi-collected-works-volume-47.pdf.

26 Mahatma Gandhi, *The Collected Works of Mahatma Gandhi Vol. 25*, p. 37, accessed on May 25, 2024, http://www.gandhiashramsevagram.org/gandhi-literature/mahatma-gandhi-collected-works-volume-25.pdf.

27 Mahatma Gandhi, *The Collected Works of Mahatma Gandhi* vol. 25, p. 91, accessed on May 25, 2024, http://www.gandhiashramsevagram.org/gandhi-literature/mahatma-gandhi-collected-works-volume-25.pdf.

28 Mahatma Gandhi, *Collected Works of Mahatma Gandhi Vol. 21*, p. 199, accessed on May 25, 2024, http://www.gandhiashramsevagram.org/gandhi-literature/mahatma-gandhi-collected-works-volume-21.pdf.

29 Mahatma Gandhi, *The Collected Works of Mahatma Gandhi Vol. 25*, p. 91, accessed on May 25, 2024, http://www.gandhiashramsevagram.org/gandhi-literature/mahatma-gandhi-collected-works-volume-25.pdf.

30 Mahatma Gandhi, *The Collected Works of Mahatma Gandhi Vol. 37*, p. 21–22, accessed on May 25, 2024, http://www.gandhiashramsevagram.org/gandhi-literature/mahatma-gandhi-collected-works-volume-37.pdf.

31 Mahatma Gandhi, *The Collected Works of Mahatma Gandhi Vol. 23*, p. 83, accessed on May 25, 2024,http://www.gandhiashramsevagram.org/gandhi-literature/mahatma-gandhi-collected-works-volume-23.pdf.

32 Aristotle, *Nicomachean Ethics*, trans. W. D. Ross (Batoche Book: Kitchener, 1999), 13, 18.

33 Aristotle, *Nicomachean Ethics*, trans. W. D. Ross (Batoche Book: Kitchener, 1999), 26, 32.

34 Aristotle, *Nicomachean Ethics*, trans. W. D. Ross (Batoche Book: Kitchener, 1999), 32.

35 Aristotle, *Nicomachean Ethics*, trans. W. D. Ross (Batoche Book: Kitchener, 1999), 28.

36 Aristotle, *Nicomachean Ethics*, trans. W. D. Ross (Batoche Book: Kitchener, 1999), 28–32.

37 Aristotle, *Nicomachean Ethics*, trans. W. D. Ross (Batoche Book: Kitchener, 1999), 27.

38 Aristotle, *Nicomachean Ethics*, trans. W. D. Ross (Batoche Book, Kitchener, 1999). Aristotle, *The Art of Rhetoric*, trans. W. Rhys Roberts (Megaphone eBooks, 2008).

39 John Saunders, “Compassion,” *Clinical Medicine* 15, no. 2 (2015): 121.

40 Aristotle, *The Art of Rhetoric*, trans. W. Rhys Roberts (Megaphone eBooks, 2008), 111–112.

41 Aristotle, *Nicomachean Ethics*, trans. W. D. Ross (Batoche Book, Kitchener, 1999), 42.

